# Pericardial Effusion Due to *Trichosporon japonicum*: A Case Report and Review of the Literature

**DOI:** 10.3390/pathogens11050598

**Published:** 2022-05-20

**Authors:** Estelle Menu, Jihane Kabtani, Johanna Roubin, Stéphane Ranque, Coralie L’Ollivier

**Affiliations:** 1 Laboratoire de Parasitologie-Mycologie, IHU Méditerranée Infection, 13385 Marseille, France; stephane.ranque@ap-hm.fr (S.R.); coralie.lollivier@ap-hm.fr (C.L.); 2 VITROME: Vecteurs-Infections Tropicales et Méditerranéennes, Service de Santé des Armées, Assistance Publique-Hôpitaux de Marseille, Institut de Recherche pour le Développement, Aix Marseille Université, 13385 Marseille, France; kabtanijihane@hotmail.com; 3 Department of Cardiovascular Critical Care Medicine, La Timone Adult Hospital, AP-HM, Aix Marseille Université, 13385 Marseille, France; johanna.roubin@ap-hm.fr

**Keywords:** *Trichosporon* *japonicum*, yeast, pericarditis

## Abstract

Invasive infections due to *Trichosporon* spp. are life-threatening opportunistic fungal infections that may affect a wide array of organs. Here, we described a case of pericardial effusion due to *Trichosporon* *japonicum* in a 42-year-old female after a heart transplantation. *T. japonicum* was isolated from the pericardial fluid, pericardial drain hole and the swab of the sternal surgery scar wound. The late mycological diagnosis due to blood culture negative, the ineffective control of pulmonary bacterial infection and the late start antifungal therapy were the contributing factors in the patient’s death.

## 1. Introduction

*Trichosporon* are emerging opportunistic basidiomycetous yeast-like organisms. Ubiquitous in the environment, they are occasionally involved in invasive fungal diseases [1]. Patients with hematological malignancies, persistent neutropenia, intravenous and urinary catheters, those who have had thoracic or abdominal surgery, and those who are immunosuppressed or pre-exposed to antifungal therapy, especially to echinocandins, are at risk of invasive trichosporonosis [2]. The most common species involved in clinical infections are *Trichosporon asahii*, followed by *T. inkin*, *T. faecale*, and *T. asteroides* [3].

In this report, we present a rare case of a *Trichosporon japonicum* infection in a 42 years old female following heart transplantation. We discussed the value of the different diagnostic tools available and performed a review of the literature on *T. japonicum* infections.

## 2. Case Presentation

### 2.1. Patient History

**Figure 1:**
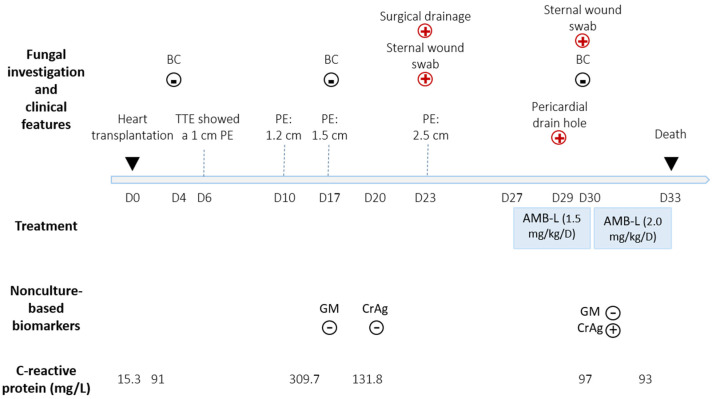
Timeline of *Trichosporon japonicum* pericarditis course. TTE: trans-thoracic echography; AMB-L: Amphotericin B liposomal; BC: Blood culture; PE: Pericardial effusion; CrAg: Cryptococcal antigen; GM: *Aspergillus* galactomannan; +: positive; −: negative; 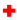: positive *Trichosporon japonicum* culture; ▪: negative *Trichosporon japonicum* culture.

A 42-year-old female was admitted at La Timone hospital (Marseille, France) to undergo a heart transplantation. She had a history of congenital cardiopathy (single ventricle) with multiple cardiac decompensation episodes, severe left ventricular dysfunction (LVEF 25%) and New York Heart Association class III dyspnea. The patient was on mechanical ventilation throughout the duration of the hospitalization, and did not develop any fever. At day 6 post transplantation, a routine trans-thoracic echography showed a 1 cm pericardial effusion (Figure 1). The pericardial effusion progressively increased to 1.2 cm at day 10, and 1.5 cm at day 17. At day 13, the patient developed an acute respiratory distress syndrome, and HSV-1 PCR was positive (in blood and bronchoalveolar lavage fluid); the patient was treated with acyclovir. The following bacteria were repeatedly cultured from respiratory samples from day 6 post transplant: *Pseudomonas aeruginosa* (one bronchial aspirate and one bronchoalveolar lavage fluid), *Stenotrophomonas maltophilia* (three bronchial aspirate, one bronchoalveolar lavage fluid and one sputum) and *Citrobacter freundii* (one bronchial aspirate and one bronchoalveolar lavage fluid). Following that, the patient was treated with an adapted antibiotic therapy, which unfortunately does not enable the control of the infection. From the first day post transplant, the patient had a supranormal white blood cell count with an average of 28 × 10^9^/L (range: 18 × 10^9^–43 × 10^9^).

### 2.2. Diagnostic Assessment and Therapeutic Intervention

**Figure 2:**
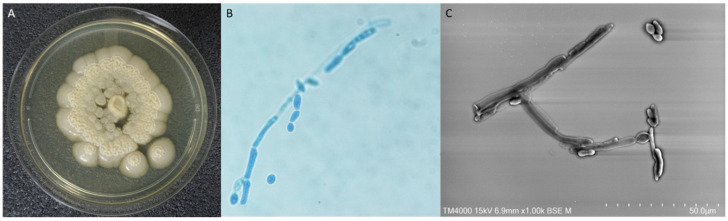
*Trichosporon japonicum* morphological features. (**A**) Colony of *Trichosporon japonicum* on Sabouraud dextrose agar media. (**B**) Fresh microscopic examination of the colonies stained with Mycetblue^®^ (Biosynex, Graffenstaden, France) ×1000 original magnification. (**C**) Scanning Electron Microscopy examination (15 KeV, lens mode 3, Scale bar 50 μm) of the colonies using the TM4000 PlusTM (Hitachi, Japan) instrument.

At day 23, the pericardial effusion became circumferential and measured 2.4 cm. The patient underwent a surgical drainage. The pericardial aspirate was purulent and grew cream-colored dry wrinkled colonies with irregular margins after 3 days (Figure 2A). Bacterial culture was negative. Fresh microscopic examination of the colonies revealed round yeast cells with blastoconidia and arthroconidia (Figure 2B,C). *Trichosporon japonicum* was identified by MALDI-TOF mass spectrometry (MicroflexLT, Bruker Daltonics GmbH, Bremen, Germany) with a Logscore value at 2.02 obtained from the standard manufacturer library, and it was deposited (strain number: IHEM 28563). DNA sequence-based identification was performed as previously described [4]. The 100% identity with the rRNA intergenic spacer IGS1 sequence (GenBank AB066426.1) of JCM8357, the type strain of *T. japonicum*, confirmed this identification. The other genomic regions rRNA Internal Transcribed Spacer 1 and 2, the D1/D2 domains of the rRNA large-subunit were less informative but in agreement with this identification. All nucleotide sequences of the present isolate were submitted to GenBank (Acc. No OM865139, OM865141 and OM897590). The NCBI BLASTn results are detailed in Supplementary Table S1. The same yeast was isolated from the swab of sternal surgery scar wound (day 23 and day 30) and pericardial drain hole (day 29). Blood culture (BacT/ALERT system, bioMerieux, Craponne, France) remain negative for the duration of the stay. *Aspergillus* galactomannan antigenaemia (Bio-Rad Laboratories, Marnes-La-Coquette, France) was negative, at day 17 and day 30. Cryptococcal serum antigen (CrAg^®^ LFA kit, IMMY, Norman, OK, USA) was negative at day 20 but positive (1:80 titer) at day 30.

The patient was treated with liposomal amphotericin B (1.5 mg/kg/D) starting from day 27. The patient developed a multiple organ failure and died at day 33.

Antifungal susceptibility testing was performed on the strain cultured from the pericardial fluid by using the colorimetric broth microdilution Sensititre Yeast-OneTM YO10 (Thermo Fisher Diagnostics, Dardilly, France) and/or the agar diffusion EtestTM (BioMérieux, Craponne, France). The following minimum inhibitory concentration (MIC) values of *T. japonicum* isolate were found: amphotericin B (YO10: 0.5 mg/L; Etest: 0.5 mg/L), micafungin (YO10: >8 mg/L), caspofungin (YO10: >8 mg/L), anidulafungin (YO10: >8 mg/L), 5-fluorocytosine (YO10: 4 mg/L), voriconazole (YO10: 0.12 mg/L; Etest: 0.125 mg/L), posaconazole (YO10: 0.25 mg/L; Etest: 0.75 mg/L), itraconazole (YO10: 0.12 mg/L), fluconazole (YO10: 4 mg/L) and isavuconazole (Etest: 0.38 mg/L).

## 3. Discussion

*Trichosporon japonicum* was first isolated in 1998 from air collected in the house of a patient with summer-type hypersensitivity pneumonitis in Japan [5]. So far, *T. japonicum* infections have been scarcely reported in the literature. A review of the English language literature in Medline by using the following keywords: “*Trichosporon japonicum*” AND “Human” (Table 1) found only five references reporting a total of six human cases of *T. japonicum* infections. These infections were documented in blood (*n* = 2) [6,7], urinary tract (*n* = 2) [8] and respiratory tract (*n* = 2) from respiratory samples [9,10]. The mean age of reported cases was 24 years (range: 8–50), the sex ratio was 1.5 and three patients died. Regarding the patients’ risk factors, two patients were kidney transplant recipients, two patients had a hematologic malignancy (AML, ALL), and similarly to our patient, one underwent cardiac surgery. No data was provided about the remaining case in whom *Trichosporon japonicum* DNA was detected in lower respiratory specimens [10]. In the present case, our patient developed a pericardial effusion following heart transplantation. The heart preservation fluid culture remained sterile, which should rule out transmission by the graft. Finally, our patient is the second case described following heart surgery and the third after solid organ transplantation.

Prompt diagnosis and timely management of trichosporonosis are essential. The gold standard diagnosis is the culture with growth between 48 and 72 h on Sabouraud medium [11] and their ability to grow on non-specific media. In the present case, the repetitive blood cultures remained negative, but the samples grew rapidly (within 72 h). *T. japonicum* was identified by the MALDI TOF MS and DNA sequencing confirmed the identification. Whereas the ITS and D1/D2 domains of rDNA are considered as the gold standard for medically important yeasts identification [12], in this present case, they could not differentiate *T. japonicum*, *T. asahii* and *T. asteroides*. The IGS region is more discriminating and has confirmed the species (Table S1). Our results confirm previous reports that the rRNA IGS1 region nucleotide sequence is the most discriminating and relevant for the precise species identification within the *Trichosporon* genus [1,11,13,14,15].

Like *Cryptococcus* spp., *Trichosporon* spp. are Basidiomycota, and cross-reactions between cryptococcal polysaccharide antigen detection and *Trichosporon* species are known [16,17]. Until now, the reported cases of cross-reactions concerned the following species: *Trichosporon cutaneum* [18,19], *Trichosporon beigelii* [16,20,21,22,23], *Trichosporon asahii* [24,25] and *Trichosporon dermatis* [26]. We present the first case of a positive cryptococcal antigen detection during a *Trichosporon japonicum* infection. Interestingly, in the literature, only Bogomin et al. [6] performed a cryptococcal antigen detection in a *T. japonicum* fungemia in a patient with a transcutaneous biventricular assist device. The latex agglutination cryptococcal capsular polysaccharide antigen test returned negative, although performed concomitantly with positive blood cultures. In our patient, the serum cryptococcal antigen using CrAg^®^ LFA kit (IMMY, Norman, OK, USA) was negative 3 days before the pericardial fluid puncture and positive 7 days after. Three situations have been reported regarding the time to serum cryptococcal antigen positivity in patients infected with the other *Trichosporon* species. Karigane et al. reported *Trichosporon asahii* fungemia in an AML patient with positive cryptococcal antigenemia 5 days before the isolation of the fungus [25], while others reported a positive cryptococcal antigen concomitantly [16] or after the yeast isolation [20,26]. Interestingly, in our patient, the cryptococcal antigen was positive in several biological fluids, such as urine or cerebrospinal fluid, all confirmed by a subsequent *Trichosporon* positive culture [19,22].

Cross-reaction between *Aspergillus* galactomannan detection and *Trichosporon* spp. have been reported [27,28]. A dual positivity of cryptococcal antigen and *Aspergillus* galactomannan has been described in case of disseminated *Trichosporon dermatis* infection [26]. In our patient, *Aspergillus* galactomannan assay in the serum at day 17 and day 30 was negative. We tested cryptococcal antigen (IMMY, Norman, OK, USA) and Platelia *Aspergillus* galactomanan (Bio-Rad Laboratories, Marnes-La-Coquette, France) in the supernatant from a *T. japonicum* culture. Both returned positive, suggesting that *T. japonicum* share common epitopes with *Cryptococcus* and *Aspergillus* cell wall components. As recommended in the ESCMID and ECMM joint clinical guidelines [28], in case of suspected invasive *Trichosporon* infection, the diagnosis should include the combined use of culture, *Aspergillus* galactomannan and cryptococcal antigen determination. It would be interesting to extend the use of these tools to the first-line diagnostic approach of invasive fungal infections.

There is no consensus on the treatment of trichosporonosis and data concerning MIC interpretation for all antifungal drugs are scarce [3]. Like in most basidiomycetes, the use of echinocandins is not recommended due to natural resistance [29]. Consistently, in our case, the isolates of *T. japonicum* showed high MIC for echinocandins and low MIC for azoles except for fluconazole. Despite data suggesting a discrepancy between in vitro and in vivo activity, susceptibility testing is still recommended for epidemiological knowledge [3,8]. Recent guidelines moderately recommend voriconazole for initial antifungal therapy, exhibiting excellent in vitro and in vivo activity against *Trichosporon* spp. [3,8]. In the present case, treatment with amphotericin B was initiated following sensitivity testing and late in the course of the disease. Therapeutic failure is thus difficult to assess.

## 4. Conclusions

*Trichosporon japonicum* infection is rare. In case of suspected invasive *Trichosporon* infection, prompt diagnosis, including the combined use of culture, *Aspergillus* galactomannan and cryptococcal antigen determination and the rapid initiation of antifungal treatment are essential.

## Figures and Tables

**Table 1:** Cases report of *Trichosporon japonicum* infections, review of literature (including the present case).

Age	Gender	Comorbidity Conditions	Clinical Presentation	Site of Positive Culture	Treatment		Outcome	Reference
					Molecule	Duration		
8	F	AML	Respiratory distress	Sputum	AMB-L (5 mg/kg/D) + ITRA (100 mg/D)	NS	Death	[9]
-	-	-	Hypersensitivity pneumonitis	BALF	-	-	-	[10]
18	F	Transcutaneous biventricular assist device	Fungemia	Blood, aortic cannula, removed left ventricular apex cuff	AMB-L + 5FC switch VORI	11 days / 6 weeks	Survival at 2 months	[6]
36	M	Kidney transplant recipient	Urinary tract infection	Urine	VORI + CASPO	NS	Survival	[8]
50	M	Kidney transplant recipient	Urinary tract infection	Urine	VORI + CASPO	15 days	Death	[8]
8	M	ALL	Fungemia	Blood	AMB (3 mg/kg/D) + VORI (8 mg/kg twice a day)	11 days	Death	[7]
42	F	Heart transplant recipient	Pericardial effusion	Pericardial fluid	AMB-L (1.5 mg/kg/D)	6 days	Death	Present case

AML: Acute Myeloid Leukemia; ALL: acute B cell lymphoblastic leukemia; F: Female; M: Male; D: day; BALF: Broncho alveolar lavage fluid; AMB-L: Liposomal amphotericin B; ITRA: itraconazole; 5FC: 5-fluorocytosine; CASPO: caspofungin; VORI: voriconazole; NS: Not Specified.

## Data Availability

Not applicable.

## Supplementary Materials

The following supporting information can be downloaded at: https://www.mdpi.com/article/10.3390/pathogens11050598/s1, **Table S1**: Molecular identification of *Trichosporon japonicum* sequencing ITS, D1/D2 and IGS genetic regions.
