# Supplementary material for: Pericardial Effusion Due to Trichosporon japonicum: A Case Report and Review of the Literature
Source: Pathogens. 2022 May 20;11(5):598. doi: 10.3390/pathogens11050598 (PMC9145057; doi:10.3390/pathogens11050598)
Supplement: Supplementary file 1 [file pathogens-11-00598-s001.zip › pathogens-1713242-supplementary.pdf]

Table S1. Molecular identification of *Trichosporon japonicum* sequencing ITS, D1/D2 and IGS genetic regions.

	**ITS**				**D1/D2**				**IGS1**			
Samples	ID	Type strain ID collection	Nucleotide alignement (identity %)	Genbank accession no.	ID	Type strain ID collection	Nucleotide alignement (identity %)	Genbank accession no.	ID	Type strain ID collection	Nucleotide alignement (identity %)	Genbank accession no.
Pericardial fluid	*Trichosporon japonicum*	CBS 8641	472/473 (99.79%)	NR_073263.1	*Trichosporon japonicum*	CBS 8641	492/493 (99.80%)	KY109955.1	*Trichosporon japonicum*	JCM8357	458/458 (100%)	AB066426.1
	Trichosporon asteroides	CBS 2481	471/473 (99.58%)	KY105727.1	*Trichosporon asahii*	CBS 2479	491/492 (99.80%)	KY109922.1	*Trichosporon asteroides*	CBS 2481	462/477 (96.86%)	EU934802.1
	Trichosporon insectorum/ faecale	CBS10422/ CBS 4828	470/473 (99.37%)	KY105746.1 / KY105736.1	*Trichosporon asteroides*	CBS 2481	491/493 (99.59%)	KY109937.1	*Trichosporon faecale*	CBS 4828	407/509 (79.96%)	KM488293.1
	Trichosporon asahii	CBS 2479	469/473 (99.15%)	KY105709.1	*Trichosporon insectorum*	CBS 10422	489/491 (99.59%)	KY109953.1	*Trichosporon asahii*	PUMCH BY15	403/505 (79.80%)	JF303013.1
Swab no. 1 (sternal surgery scar wound)	*Trichosporon insectorum*	CBS 10422	425/425 (100%)	KY105746.1	*Trichosporon japonicum*	CBS 8641	547/547 (100%)	KY109955.1	*Trichosporon japonicum*	JCM8357	448/448 (100%)	AB066426.1
	Trichosporon faecale	CBS 4828	425/425 (100%)	KY105736.1	*Trichosporon asteroides*	CBS 2481	546/547 (99.82%)	KY109937.1	*Trichosporon asteroides*	CBS 2481	453/468 (96.79%)	EU934802.1
	Trichosporon japonicum	CBS 8641	425/425 (100%)	NR_073263.1	*Trichosporon asahii*	CBS 2479	545/547 (99.63%)	KY109922.1	*Trichosporon faecale*	CBS 4828	395/479 (79.48%)	KM488293.1
	Trichosporon asahii	CBS 2479	423/423 (100%)	AB018013.1	*Trichosporon insectorum*	ATCC MYA-4361	542/547 (99.09%)	NG_042467.1	*Trichosporon asahii*	PUMCH BY15	391/493 (79.31%)	JF303013.1
Swab no. 2 (sternal surgery scar wound)	*Trichosporon insectorum*	CBS 10422	427/427 (100%)	KY105746.1	*Trichosporon japonicum*	CBS 8641	486/486 (100%)	KY109955.1	*Trichosporon japonicum*	JCM8357	470/470 (100%)	AB066426.1
	Trichosporon faecale	CBS 4828	427/427 (100%)	KY105736.1	*Trichosporon asahii*	CBS 2479	486/486 (100%)	KY109922.1	*Trichosporon asteroides*	CBS 2481	490/506 (96.84%)	EU934802.1
	Trichosporon japonicum	CBS 8641	427/427 (100%)	NR_073263.1	*Trichosporon insectorum*	CBS 10422	485/486 (99.79%)	KY109953.1	*Trichosporon faecale*	CBS 4828	432/534 (80.90%)	KM488293.1
	Trichosporon asahii/asteroides	CBS2479 / CBS 2481	426/427 (99.77%)	KY105709.1 / KY105727.1	*Trichosporon asteroides*	CBS 2481	485/486 (99.79%)	KY109937.1	*Trichosporon asahii*	PUMCH BY15	427/529 (80.72%)	JF303013.1
Swab no. 3 (Subcutaneous mediastinal sample)	*Trichosporon japonicum*	CBS 8641	483/483 (100%)	NR_073263.1	*Trichosporon japonicum*	CBS 8641	496/496 (100%)	KY109955.1	*Trichosporon japonicum*	JCM8357	455/455 (100%)	AB066426.1
	Trichosporon asteroides	CBS 2481	482/483 (99.79%)	KY105727.1	*Trichosporon asahii*	CBS 2479	496/496 (100%)	KY109922.1	*Trichosporon asteroides*	CBS 2481	461/476 (96.85%)	EU934802.1
	Trichosporon insectorum/faecale	CBS10422/ CBS 4828	481/483 (99.59%)	KY105746.1 / KY105736.1	*Trichosporon insectorum*	CBS 10422	495/496 (99.80%)	KY109953.1	*Trichosporon faecale*	CBS 4828	403/505 (79.80%)	KM488293.1

	Trichosporon asahii	CBS 2479	480/483 (99.38%)	KY105709.1	*Trichosporon asteroides*	CBS 2481	495/496 (99.80%)	KY109937.1	*Trichosporon asahii*	PUMCH BY15	399/501 (79.64%)	JF303013.1

ID: identification; ITS: internal transcribed spacer; D1/D2: domains of the ribosomal DNA large-subunit; IGS: intergenic spacer; no.: number
